# Alcohol-antiretroviral interactive toxicity beliefs as a potential barrier to HIV pre-exposure prophylaxis among men who have sex with men

**DOI:** 10.7448/IAS.20.1.21534

**Published:** 2017-07-17

**Authors:** Seth C Kalichman, Lisa Eaton

**Affiliations:** ^a^ Center for Health, Intervention, and Prevention, University of Connecticut, Storrs, CT, USA

**Keywords:** HIV prevention, pre-exposure prophylaxis, substance use, men who have sex with men

## Abstract

**Introduction**: Pre-exposure prophylaxis (PrEP) offers as much as 90% protection against HIV transmission. However, the effectiveness of PrEP depends on uptake and adherence to even intermittent dosing. Along with intoxication leading to unintentional non-adherence, believing that alcohol mixed with pharmaceuticals is harmful (i.e., interactive toxicity beliefs) may lead to poor uptake and intentional non-adherence.

**Methods**: HIV-negative sexually active men who have sex with men (*N* = 272) at a large Gay Pride event in Atlanta, GA, completed anonymous surveys of demographic characteristics, sexual behaviour, alcohol use and PrEP-related alcohol interactive toxicity beliefs.

**Results**: A total of 118 (43%) men surveyed had two or more male sex partners and condomless anal sex in the previous six months. Alcohol use was reported by over 90% of men and it was common for participants to believe that mixing alcohol and antiretrovirals is toxic; 75% endorsed at least one interactive toxicity belief. Among the 118 men who had engaged in condomless anal sex and had multiple sex partners, one in three stated that they were not interested in PrEP and men not interested in PrEP were significantly more likely to binge drink and hold interactive toxicity beliefs.

**Conclusions**: These results mirror studies that find interactive toxicity beliefs are a potent predictor of intentional antiretroviral non-adherence among people living with HIV and suggest interactive toxicity beliefs may impede PrEP uptake and adherence. Messages to increase PrEP awareness and adherence may also take steps to counter erroneous beliefs about mixing alcohol with antiretrovirals in the context of PrEP.

## Introduction

Antiretroviral (ARV) medications are now at the forefront of HIV prevention, with pre-exposure prophylaxis (PrEP) offering the potential for protection against HIV transmission [1]. Most compelling are results of the iPrEx trial, which indicated that ARV exposure, specifically Truvada – the combination of emtricitabine (FTC) and tenofovir disoproxil fumarate (TDF) – resulted in a 44% reduction in HIV incidence. Among the 100 men in iPrEx who became HIV infected, 36 received PrEP. However, ARV was detected in only 9% of infected men, indicating that PrEP resulted in over 90% protection. The difference between the 44% protection in overall findings versus 90% protection in the sub-analyses lies in adherence to a daily PrEP regimen [2–4]. Moreover, patterns of pill taking may strongly influence PrEP efficacy, with protection dropping to 34% with consecutive days of non-adherence [5]. As PrEP is scaled-up and targeted to populations at greatest risk for HIV infection, increased attention is needed to assure optimal levels of PrEP adherence [6].

Among the most robust threats to ARV adherence is substance use [7,8]. For example, individuals who use stimulants perceive greater concern that substance use can interfere with PrEP [9]. Furthermore, these concerns may impede uptake of PrEP among substance using individuals at risk for HIV, including men who have sex with men (MSM) [10]. All drugs of abuse may impede uptake and adherence to PrEP [11], with alcohol use posing the most common challenge [12,13]. Alcohol use impacts ARV adherence through multiple mechanisms, including disrupting memory, attention and sleep [14,15]. In parallel to therapeutic use of ARV, alcohol use is also associated with PrEP non-adherence [16]. While alcohol intoxication clearly leads to unintentional non-adherence, alcohol drinkers may also intentionally forego taking ARV because they believe that mixing alcohol with pharmaceuticals is harmful, referred to as interactive toxicity beliefs [17,18]. As many as one in four people living with HIV who drink alcohol stop taking ARV when drinking because they hold interactive toxicity beliefs [19,20]. These beliefs are common among people living with HIV, with more than half of HIV positive drinkers who take ARV stopping treatment when they are drinking [20]. Interactive toxicity beliefs are prevalent and directly associated with intentional non-adherence to the therapeutic use of ARV [18,21,22]. While these same beliefs may lead to hesitation to start PrEP as well as PrEP non-adherence, we are not aware of any research examining the potential for interactive toxicity beliefs to impede PrEP use.

Because alcohol use is a robust predictor of sexual risks in populations vulnerable to HIV, drinking is likely prevalent among individuals who are candidates for PrEP [9]. Interactive toxicity beliefs directed towards PrEP may therefore risk PrEP refusal and intentional PrEP non-adherence. The current study focuses on PrEP interactive toxicity beliefs among MSM; 67% of US HIV infections occur among MSM and PrEP promotion in the US is most visibly targeted to this population [23]. We examined the degree to which MSM who may be appropriate for PrEP hold alcohol interactive toxicity beliefs and whether these beliefs may impede PrEP use.

## Methods

### Participants, setting and procedures

Participants were 394 men surveyed at the Atlanta Gay Pride Festival in October 2015. Surveys were collected using venue sampling procedures that have been reported in previous studies [10,24]. Participants were told that the survey was about health behaviours, contained personal questions about their behaviour, was anonymous, and would take 15 minutes to complete. Eighty per cent of men approached agreed to complete the survey. Participant names were not obtained on the surveys. Participants were offered $5 for completing the survey with an additional $5 donated to a local AIDS service organization. Participants provided a first name and last initial on receipts for payments unassociated with the surveys. We examined the surveys for completeness and checked for potential repeated surveys using the receipts. No repeat surveys were identified. The survey for this study is available as supplementary materials.

### Ethical approval and consent

All participants were age 18 and older and provided verbal informed consent in compliance with conducting self-administered anonymous surveys.

### Measures

#### Demographic characteristics

Participants were asked their age, years of education, income and race. Openness about sexual orientation was assessed with the item “How out are you about your sexual orientation?” with responses “not out about sexual orientation”, “sometimes out about sexual orientation” and “out about sexual orientation”. Participants also reported whether they had been tested for HIV and the result of their most recent HIV test.

#### Sexual behaviour

Participants reported the number of times they had engaged in anal intercourse as the insertive and receptive partner, with and without condoms in the past six months. Participants also recorded the number of sexual partners with whom they had engaged in each behaviour. Open response formats were used for the sexual behaviour measures to reduce response bias and to minimize measurement error [25]. Proportion of condom use during anal intercourse was calculated with the formula *frequency of condom protected acts/total frequency of acts*. Measures similar to these have been found reliable in self-reported sexual behaviour assessments [25,26].

#### Substance use

Three alcohol use items were adapted from the consumption scale of the Alcohol Use Disorders Identification Test [27,28], and were used to assess frequency and quantity of alcohol use with reference to current drinking. Specifically, we asked “how often do you have a drink containing alcohol?”, with responses “never”, “monthly or less”, “2–4 times a month”, “2–3 times a week” or “more than 4 times week”. Responses were recoded to represent none, monthly or at least weekly drinking. Quantity of alcohol consumption was assessed in terms of numbers of drinks in a typical drinking session, specifically “how may drinks containing alcohol do you have on a typical day when you are drinking?” (response categories shown in results). Finally, binge drinking was assessed using the item “How often do you have 6 or more drinks on one occasion?”, responses were “never”, “less than monthly”, “monthly”, “weekly” and “daily or almost daily” recoded to represent never, less than monthly and monthly or more often binge drinking. Participants were also asked whether they used cannabis, powder or crack cocaine, or other non-prescription drugs in the past six months coded dichotomously, Yes/No.

#### PrEP awareness

Participants were asked about their awareness and interest in PrEP. These measures specified the use of Truvada, the brand name for FTC/TDF, which is FDA approved for PrEP use. Items were adapted from previous research on PrEP awareness among MSM [29]. First, we provided participants with a standard definition of PrEP. Specifically, as part of the instructions the survey read “PrEP (pre-exposure prophylaxis) is when an HIV-negative person takes anti-HIV medications, also known as antiretrovirals and more specifically Truvada, BEFORE HAVING SEX to prevent HIV infection”. Participants responded Yes or No to each of the following: “Have you ever heard of PrEP?”, “Do you know anyone who is taking PrEP?”, “Are you currently taking PrEP?” and “Would you be interested in taking PrEP?”.

#### PrEP-related interactive toxicity beliefs

Truvada can itself have mild side effects, which can be amplified when mixed with alcohol. The Truvada pharmaceutical packaging states “Truvada may cause dizziness. This effect may be worse if you take it with alcohol or certain medicines. Use Truvada with caution. Do not drive or perform other possibly unsafe tasks until you know how you react to it”. Thus, mixing Truvada with alcohol is not completely benign, but does not raise significant toxicity concerns, and there is no warning of health complications resulting from mixing Truvada with alcohol. We used five items to assess PrEP-related alcohol interactive toxicity beliefs that directly reflect beliefs regarding the potential health hazards from mixing alcohol with Truvada. Items were adapted from previous studies of therapeutic use of ARV and alcohol interactive toxicity beliefs [18,21]. Items are shown in the results and were responded to using a 4-point scale, from “Strongly disagree” to “Strongly agree”. Responses were dichotomized as agreement and disagreement for ease of interpretation. Interactive toxicity belief scores were also computed by taking the mean for the full range of responses, *α* = .89.

### Data quality assurances and data analyses

All surveys were examined for inconsistencies and invalid responses that were treated as missing values (less than 5%), resulting in slightly different “n”s for analyses. For the purposes of this study, all main analyses were restricted to men who were sexually active with a male partner in the past six months and did not report having tested HIV positive. Within this sample, we used the following criteria to define men who were potentially appropriate for PrEP, or PrEP candidates; self-identified men who reported two or more male sex partners in the previous six months and at least one occasion of condomless anal intercourse. These criteria are narrower than the CDC PrEP guidelines, which include long-term serodiscordant partnerships and recent sexually transmitted infections [30]. We therefore focused on a subset of MSM who engage in behaviour well recognized as high risk for HIV infection and targeted by PrEP awareness campaigns [31]. We first compared men who were PrEP candidates (*n* = 118) to their non-candidate sexually active counterparts (*n* = 154). Second, we restricted the sample to only those participants who were defined as PrEP candidates and were currently drinking alcohol (*n* = 108) and compared men who were interested or currently taking PrEP (*n* = 73) to men who were not interested in PrEP (*n* = 35). This sub-analysis concentrated on participants who reported current alcohol use, the most relevant individuals in terms of questions of alcohol interactive toxicity beliefs and PrEP. Finally, we tested a multivariable model of interactive toxicity beliefs as predictors of interest in PrEP among MSM who drink and were candidates for PrEP. Analyses were performed using logistic regressions reporting odds ratios (OR) with 95% confidence intervals (95% CI). Data analyses were performed using SPSS, version 23.

## Results

A total of 394 men completed surveys, of which 66 (17%) were HIV positive and 56 (14%) reported no same sex behaviour in the previous six months. The final sample therefore consisted of 272 sexually active MSM who had not tested HIV positive. A total of 118 (43%) men engaged in condomless anal sex and had multiple sex partners, defined here as PrEP candidates. The median number of condomless anal intercourse acts during that time period was four, ranging from 1 to 102. PrEP candidates had lower incomes, were younger, less likely to have health insurance, and less likely to have a healthcare provider, had significantly more male partners, were more likely to know someone taking PrEP and were more interested in taking PrEP (see Table 1).

**Table 1.** Characteristics of sexually active MSM who are not and who are PrEP candidates

	Not a PrEP candidate (*N* = 154)		PrEP candidate (*N* = 118)			
Characteristic	*N*	%	*N*	%	OR	95% CI
Race						
Caucasian	88	57	64	55	Reference	
African American	42	27	35	30	1.33	0.47–3.79
Hispanic/Latino	13	8	12	10	1.52	0.51–4.54
Other race	11	7	6	5	1.69	0.48–6.00
Income <$15,000	25	16	32	27		
$16,000–$30,000	29	19	29	25		
$31,000–$45,000	30	20	34	21		
$46,000–$60,000	27	18	13	11		
>$60,000	42	27	19	16	0.80*	0.69–0.92
Employed	123	80	94	80	1.01	0.55–1.84
Has health insurance	127	83	86	73	0.55*	0.30–0.98
Has healthcare provider	132	87	90	76	0.49*	0.26–0.92
Not openly Gay	8	5	1	1	Reference	
Sometimes openly Gay	40	27	29	25	0.14	0.02–1.19
Always openly Gay	102	68	87	74	0.85	0.49–1.48
Heard of PrEP	98	64	87	73	1.60+	0.94–2.71
Knows someone taking PrEP	53	34	63	53	2.18**	1.33–3.56
Currently taking PrEP	10	7	14	12	1.95	0.83–4.57
Interested in taking PrEP	61	40	77	66	2.90**	1.76–4.79
	M	SD	M	SD		
Age (years)	37.1	12.8	30.6	12.1	0.95**	0.93–0.97
Education (years)	14.4	2.2	14.5	2.1	1.02	0.92–1.14
Number male partners	2.6	4.3	6.7	8.1	1.19**	1.11–1.29
Condomless anal sex	10.2	29.8	9.1	14.6	0.99	0.98–1.01
Percentage of condom use-anal intercourse	46.5	45.1	38.1	30.2	0.56	0.29–1.09

Note: OR = odds ratio; CI = confidence interval; MSM = men who have sex with men; PrEP = pre-exposure prophylaxis; +*p* < .10, *p < .05, **p < .01.

### Substance use among PrEP candidates and non-candidates

The majority of men (91%) reported current alcohol use, with 47% drinking lightly (1–2 drinks per session). However, nearly two out of three men reported binge drinking. There was a trend towards PrEP candidates being more likely to binge drink than those who were not PrEP candidates. PrEP candidates were also more likely to report recent cannabis and other drug use (see Table 2).

**Table 2.** Substance use and PrEP-related interactive toxicity beliefs among sexually active MSM who are not and who are PrEP candidates

	Not a PrEP candidate (*N* = 154)		PrEP candidate (*N* = 118)			
Characteristic	*N*	%	*N*	%	OR	95% CI
Frequency of alcohol use						
None	16	11	9	8	Reference	
Monthly	77	50	61	52	0.72	0.29–1.76
At least weekly	60	39	47	40	1.01	0.61–1.68
Quantity of alcohol use						
Does not drink	20	13	12	10	Reference	
1–2 drinks	79	52	49	42	1.20	0.19–7.57
3–4 drinks	33	22	31	27	1.24	0.22–7.02
5–6 drinks	15	10	16	13	1.88	0.32–10.99
7+ drinks	6	3	9	8	2.13	0.34–13.40
Binge drinking						
Does not binge drink	69	45	34	29	Reference	
Binge drinks less than monthly	48	31	52	44	0.57+	0.30–1.06
Binge drinks monthly or more	37	24	32	27	1.25	0.67–2.31
**Drug use in past six months**						
Cannabis use	49	32	55	57	1.89**	1.13–3.07
Cocaine use	13	9	11	10	1.11	0.48–2.58
Other non-prescription drug use	4	3	15	13	5.46**	1.76–16.92
**PrEP interactive toxicity beliefs**						
Alcohol and PrEP should never be mixed	100	65	68	57	0.72	0.43–1.19
A person should stop taking PrEP if they are drinking	71	47	44	37	0.71	0.43–1.16
Alcohol interferes with PrEP so it will not work right	78	53	51	45	0.72	0.44–1.19
Mixing alcohol with HIV medications like PrEP is dangerous	87	59	60	53	0.78	0.47–1.28
Drinking alcohol while taking PrEP is toxic to the body’s system	84	56	57	50	0.77	0.47–1.26

Note: OR = odds ratio; CI = confidence interval; MSM = men who have sex with men; PrEP = pre-exposure prophylaxis; +*p* < .07,  **p < .01.

### Interactive toxicity beliefs among PrEP candidates and non-candidates

More than 75% of participants endorsed at least one PrEP-related alcohol interactive toxicity belief, with 61% (168/272) of the sample stating that alcohol and PrEP should never be mixed and 42% (115/272) stating that a person should stop taking PrEP if they are drinking (see Table 2). More than half of the sample endorsed three or more PrEP-related alcohol interactive toxicity beliefs, with an average of 2.6 (SD = 0.85) interactive toxicity beliefs endorsed. The prevalence of alcohol interactive toxicity beliefs was therefore high, and there were no significant differences between PrEP candidates and non-candidates in their rates of endorsing interactive toxicity beliefs.

### Interactive toxicity beliefs among PrEP candidates who drink and are not or are interested in taking PrEP

Among the 118 men who were candidates for PrEP, ten did not report current alcohol use and were removed from these analyses. For the 108 PrEP candidates who currently used alcohol, 73 (66%) were interested in using PrEP (see Table 3). With respect to binge drinking, men who were interested in PrEP were more likely to binge drink on monthly or less frequently basis but were less likely to binge drink monthly or more often. Thus, men interested in PrEP were more likely to be infrequent binge drinkers, but not frequent binge drinkers. Nearly all individuals interested in PrEP (91%) had previously heard about PrEP compared to 37% of those not interested. In addition, 67% of interested persons knew someone taking PrEP compared to 23% of the non-interested.

**Table 3.** Substance use and PrEP-related interactive toxicity beliefs among sexually active MSM who are candidates for PrEP and drink alcohol who are not and who are interested in PrEP

	Not interested in PrEP (*N* = 35)			Interested in PrEP (*N* = 73)		Unadjusted	
Characteristic		*N*	%	*N*	%	OR	95% CI
**Current alcohol use**							
Frequency of alcohol use							
Monthly		16	46	44	61	Reference	
At least weekly		19	54	28	39	0.83	0.53–1.28
Quantity of alcohol use							
1–2 drinks		15	43	37	50	Reference	
3–4 drinks		12	34	19	26	1.20	0.26–5.43
5–6 drinks		5	14	11	15	0.79	0.16–3.77
7+ drinks		3	9	6	9	1.10	0.19–6.28
Binge drinking							
Does not binge drink		7	20	16	22	Reference	
Binge drinks less than monthly		11	31	41	57	2.59	0.83–7.99
Binge drinks monthly or more		17	49	15	21	4.22**	1.61–11.05
Cannabis		15	43	38	53	1.49	0.66–3.36
Cocaine		4	11	7	10	0.83	0.23–3.06
Other non-prescription drugs		5	14	10	14	0.96	0.30–3.08
Heard of PrEP 13			37	66	91	18.61**	6.31–54.87
Knows someone taking PrEP 8			23	48	67	6.75**	2.66–17.08
Currently taking PrEP 0				12	17	n/a	
PrEP interactive toxicity beliefs							
Alcohol and PrEP should never be mixed		21	62	43	61	0.99	0.42–2.29
A person should stop taking PrEP if he/she is drinking		19	58	21	30	0.32**	0.14–0.76
Alcohol interferes with PrEP so it will not work right		21	64	26	37	0.34**	0.14–0.82
Mixing alcohol with HIV medications like PrEP is dangerous		22	68	34	49	0.48	0.21–1.15
Drinking alcohol while taking PrEP is toxic to the body’s system		22	67	29	42	0.36**	0.15–0.80
Number beliefs endorsed							
0		7	21	23	33		
1		1	3	8	11		
2		5	14	9	13		
3		2	6	8	11		
4–5		19	56	22	31	0.76**	0.58–0.97
Mean (SD) beliefs score		2.86	0.77	2.40	0.95	0.55**	0.33–0.89

Note: OR = odds ratio; CI = confidence interval; MSM = men who have sex with men; PrEP = pre-exposure prophylaxis;  **p < .01.

Overall, more than three in four participants who were alcohol-using PrEP candidates endorsed at least one interactive toxicity belief. PrEP candidates who were not interested in using PrEP endorsed more PrEP-related alcohol interactive toxicity beliefs. Men who were not interested in PrEP were more likely to believe that a person should stop taking PrEP when drinking, and that mixing PrEP with alcohol renders PrEP less effective and toxic. However, more than two-thirds of men interested in PrEP still endorsed at least one interactive toxicity belief, with 30% stating that PrEP should be stopped when drinking (see Table 3).

The multivariable logistic regression model predicting PrEP interest from interactive toxicity beliefs among alcohol using PrEP candidates, controlling for age, race, years of education, employment status, income, number of male sex partners, knowing someone taking PrEP, frequency of alcohol use and binge drinking was significant, *X*^2^ (10) = 40.20, *p* < .01. Men interested in PrEP were significantly more likely to know someone taking PrEP, OR = 17.09, *p* < .01, 95% CI = 3.70–78.90, less likely to drink binge, OR = 0.34, *p* < .01, 95% CI = 0.16–0.73, and were less likely to endorse alcohol interactive toxicity beliefs, OR = 0.43, *p* < .05, 95% CI = 0.19–0.93.

### Interactive toxicity beliefs among men taking PrEP

A total of 24 participants, 9% of the sample and 20% of men who we defined as PrEP candidates, indicated that they were currently taking PrEP. Alcohol use was common among PrEP users, with 21 (88%) reporting current alcohol use and 13 (54%) binge drinking. PrEP-related alcohol interactive toxicity beliefs among men taking PrEP mirrored those observed in the larger sample with 50% of men taking PrEP endorsing at least one interactive toxicity belief and one-in-four endorsing multiple beliefs. One third of men taking PrEP indicated that alcohol and PrEP should never be mixed.

## Discussion

This is the first study that we are aware of that examines interactive alcohol toxicity beliefs in a population targeted for HIV PrEP. Consistent with a growing body of research that shows beliefs that mixing alcohol with ARV leads to intentional non-adherence among some alcohol and drug users living with HIV [20,21,32,33], we found that interactive toxicity beliefs may impede PrEP uptake and adherence. A majority of men surveyed held alcohol-PrEP interactive toxicity beliefs, with three out of four endorsing at least one belief that mixing alcohol and ARV is dangerous. Among men who were PrEP candidates, interactive toxicity beliefs were more prevalent among men who were not interested in PrEP than those men who were interested. In a multivariable model knowing someone who has taken PrEP, binge drinking and interactive toxicity beliefs differentiated PrEP candidates who were and were not interested in PrEP. Thus, it is possible that binge drinking and alcohol interactive toxicity beliefs contribute to diminished interest in PrEP, while knowing someone who uses PrEP has the opposite effect. In addition, two out of three PrEP candidates who are interested in PrEP endorsed interactive toxicity beliefs that are known to predict intentional non-adherence among people living with HIV [21]. These results suggest that alcohol interactive toxicity beliefs are a potential barrier to PrEP uptake and adherence, particularly among men who drink in greater quantities.

The current findings should be considered in light of the study methodological limitations. This study was a cross-sectional survey with a convenience sample of men attending a large gay pride event in a city in the southeastern USA. It is likely that our sample over-represents men who are open about their sexual orientation and connected to the lesbian, gay, bisexual and transgender  (LGBT) community. Our sample size was also relatively small in size with some analyses based on sparse cells and large confidence intervals. In addition, we did not use current CDC guidelines to define PrEP candidates. Rather, we used a narrower definition based on multiple partners and condomless anal sex. Our survey also relied on self-reports of socially sensitive behaviours. Although using an anonymous procedure minimized the potential influences of social desirability, studies such as ours can still yield biased data, and these biases must be considered when interpreting our findings. Another potential constraint on our results is the risk for individuals to complete multiple surveys for the incentive payment. Although we had a procedure for protecting against repeat surveys, we cannot be certain this did not occur. Our study also focused on alcohol use and alcohol-related beliefs. We recognize the potential for interactive toxicity beliefs in relation to other drugs and PrEP use. The association between alcohol and other drugs may influence interactive toxicity beliefs and should be examined in future research. Acknowledging these limitations, we believe that our findings have important implications for implementing and scaling-up PrEP.

Scaling-up PrEP has the potential to alter the course of HIV epidemics, but faces multiple challenges including increasing PrEP awareness and increasing access to health services for the uninsured [10,29]. Interactive toxicity beliefs are prevalent and may stem from an over-generalization of the potential mild interactions of Truvada and alcohol as well as recommendations to not drink when taking any medications that adversely interact with alcohol [34]. Medication adherence is hampered by concerns about side effects, dependence, and adverse outcomes [35]. In addition, PrEP carries added concerns about stigmas that are assumed with sexual risks [36]. Along with these concerns, providers should assess and directly address alcohol interactive toxicity beliefs among PrEP candidates. Failure to discuss the safety of alcohol use while taking PrEP will leave erroneous beliefs unchecked and risk PrEP refusal and intentional non-adherence.

## Conclusions

The same interactive toxicity beliefs that predict intentional ARV non-adherence among people living with HIV were found to potentially impede PrEP uptake and adherence. Messages aimed to increase PrEP awareness and adherence may take proactive steps to counter erroneous beliefs about mixing alcohol with ARV in the context of PrEP.

## References

[1] Grant RM, Lama JR, Anderson PL, McMahan V, Liu AY, Vargas L, et al Preexposure chemoprophylaxis for HIV prevention in men who have sex with men. N Engl J Med. 2010;363(27):2587–8. 21091279 10.1056/NEJMoa1011205 PMC3079639

[2] Auerbach JD. The iPrEx results: lifting hopes, raising questions. Beta. 2010;22(4):47–49. 21591604

[3] Haberer JE, Bangsberg DR, Baeten JM, Curran K, Koechlin F, Amico KR, et al Defining success with HIV pre-exposure prophylaxis: a prevention-effective adherence paradigm. Aids. 2015;29(11):1277–85. 26103095 10.1097/QAD.0000000000000647 PMC4480436

[4] Defechereux PA, Mehrotra M, Liu AY, McMahan VM, Glidden DV, Mayer KH, et al Depression and oral FTC/TDF pre-exposure prophylaxis (PrEP) among men and transgender women who have sex with men (MSM/TGW). AIDS Behav. 2016 7;20(7):1478–88. 26078115 10.1007/s10461-015-1082-2 PMC4903104

[5] Dimitrov DT, Masse BR, Donnell D PrEP adherence patterns strongly affect individual HIV risk and observed efficacy in randomized clinical trials. J Acquir Immune Defic Syndr. 2016;72(4):444–51. 26990823 10.1097/QAI.0000000000000993 PMC4925182

[6] Smith DK, Herbst JH, Rose CE Estimating HIV protective effects of method adherence with combinations of preexposure prophylaxis and condom use among African American men who have sex with men. Sex Transm Dis. 2015;42(2):88–92. 25585067 10.1097/OLQ.0000000000000238

[7] Kasserra C, Assaf M, Hoffmann M, Li Y, Liu L, Wang X, et al Pomalidomide: evaluation of cytochrome P450 and transporter-mediated drug-drug interaction potential in vitro and in healthy subjects. J Clin Pharmacol. 2015;55(2):168–78. 25159194 10.1002/jcph.384

[8] Kumar S, Rao PS, Earla R, Kumar A Drug-drug interactions between anti-retroviral therapies and drugs of abuse in HIV systems. Expert Opin Drug Metab Toxicol. 2015;11(3):343–55. 25539046 10.1517/17425255.2015.996546 PMC4428551

[9] Oldenburg CE, Mitty JA, Biello KB, Closson EF, Safren SA, Mayer KH, et al Differences in attitudes about HIV pre-exposure prophylaxis use among stimulant versus alcohol using men who have sex with men. AIDS Behav. 2016;20(7):1451–60. 26462669 10.1007/s10461-015-1226-4 PMC4833721

[10] Eaton LA, Driffin DD, Smith H, Conway-Washington C, White D, Cherry C Psychosocial factors related to willingness to use pre-exposure prophylaxis for HIV prevention among black men who have sex with men attending a community event. Sex Health. 2014;11(3):244–51. 25001553 10.1071/SH14022 PMC4331017

[11] Biello KB, Oldenburg CE, Mitty JA, Closson EF, Mayer KH, Safren SA, et al The “safe sex” conundrum: anticipated stigma from sexual partners as a barrier to PrEP use among substance using MSM engaging in transactional sex. AIDS Behav. 2016 [Epub ahead of print]. 10.1007/s10461-016-1466-y PMC5624223 27351194

[12] Tran BX, Nguyen LT, Do CD, Nguyen QL, Maher RM Associations between alcohol use disorders and adherence to antiretroviral treatment and quality of life amongst people living with HIV/AIDS. BMC Public Health. 2014;14:27. 24411007 10.1186/1471-2458-14-27 PMC3893525

[13] Kalichman SC, Grebler T, Amaral CM, McNerney M, White D, Kalichman MO, et al Viral suppression and antiretroviral medication adherence among alcohol using HIV-positive adults. Int J Behav Med. 2014;21(5):811–20. 24085706 10.1007/s12529-013-9353-7 PMC4283596

[14] Waldrop-Valverde D, Ownby RL, Wilkie FL, Mack A, Kumar M, Metsch L Neurocognitive aspects of medication adherence in HIV-positive injecting drug users. AIDS Behav. 2006;10(3):287–97. 16485072 10.1007/s10461-005-9062-6

[15] Cohn SE, Jiang H, McCutchan JA, Koletar SL, Murphy RL, Robertson KR, et al Association of ongoing drug and alcohol use with non-adherence to antiretroviral therapy and higher risk of AIDS and death: results from ACTG 362. AIDS Care. 2011;23(6):775–85. 21293986 10.1080/09540121.2010.525617 PMC3095689

[16] Van der Elst EM, Mbogua J, Operario D, Mutua G, Kuo C, Mugo P, et al High acceptability of HIV pre-exposure prophylaxis but challenges in adherence and use: qualitative insights from a phase I trial of intermittent and daily PrEP in at-risk populations in Kenya. AIDS Behav. 2013;17(6):2162–72. 23080358 10.1007/s10461-012-0317-8 PMC3690654

[17] Kalichman SC, Amaral CM, White D, Swetsze C, Pope H, Kalichman MO, et al Prevalence and clinical implications of interactive toxicity beliefs regarding mixing alcohol and antiretroviral therapies among people living with HIV/AIDS. AIDS Patient Care STDs. 2009;23(6):449–54. 19413503 10.1089/apc.2008.0184 PMC2856434

[18] Kalichman SC, Kalichman MO, Cherry C, Hoyt G, Washington C, Grebler T, et al Intentional medication nonadherence because of interactive toxicity beliefs among HIV-positive active drug users. J Acquir Immune Defic Syndr. 2015;70(5):503–09. 26226250 10.1097/QAI.0000000000000776 PMC4648658

[19] Sankar A, Wunderlich T, Neufeld S, Luborsky M Sero-positive African Americans’ beliefs about alcohol and their impact on anti-retroviral adherence. AIDS Behav. 2007;11(2):195–203. 16799842 10.1007/s10461-006-9144-0 PMC4216565

[20] Kalichman SC, Amaral CM, White D, Swetsze C, Kalichman MO, Cherry C, et al Alcohol and adherence to antiretroviral medications: interactive toxicity beliefs among people living with HIV. J Assoc Nurses AIDS Care. 2012;23(6):511–20. 22421097 10.1016/j.jana.2011.11.005 PMC4271641

[21] Kalichman SC, Grebler T, Amaral CM, McNerey M, White D, Kalichman MO, et al Intentional non-adherence to medications among HIV positive alcohol drinkers: prospective study of interactive toxicity beliefs. J Gen Intern Med. 2013;28(3):399–405. 23065532 10.1007/s11606-012-2231-1 PMC3579979

[22] Altice FL, Mostashari F, Friedland GH Trust and the acceptance of and adherence to antiretroviral therapy. J Acquir Immune Defic Syndr. 2001;28(1):47–58. 11579277 10.1097/00042560-200109010-00008

[23] CDC HIV among gay and bisexual men. 2016 Available from: http://www.cdc.gov/hiv/group/msm/

[24] Kalichman SC, Eaton L, White D, Cherry C, Pope H, Cain D, et al Beliefs about treatments for HIV/AIDS and sexual risk behaviors among men who have sex with men, 1997-2006. J Behav Med. 2007;30(6):497–503. 17690973 10.1007/s10865-007-9123-6 PMC2937187

[25] Schroder K, Carey MP, Vanable P Methodological challenges in research on sexual risk behavior: I item content, scaling, and data analytic options. Ann Behav Med. 2003;26:104–23. 14534027 10.1207/s15324796abm2602_02 PMC2452993

[26] Catania JA, Gibson D, Chitwood D, Coates TJ Methodological problems in AIDS behavioral research: influences on measurement error and participation bias in studies of sexual behavior. Psychol Bull. 1990;108:339–62. 2270232 10.1037/0033-2909.108.3.339

[27] Maisto SA, Conigliaro J, McNeil M, Kraemer K, Kelley ME An empirical investigation of the factor structure of the AUDIT. Psychol Assess. 2000;12(3):346–53. 11021159 10.1037//1040-3590.12.3.346

[28] Saunders JB, Aasland OG, Babor TF, DeLaFuente JR, Grant M Development of the alcohol use disorders identification test (AUDIT): WHO collaborative project on early detection of persons with harmful alcohol consumption II. Addictions. 1993;88(6):791–804. 10.1111/j.1360-0443.1993.tb02093.x 8329970

[29] Eaton LA, Driffin DD, Bauermeister J, Smith H, Conway-Washington C Minimal awareness and stalled uptake of pre-exposure prophylaxis (PrEP) among at risk, HIV-negative, black men who have sex with men. AIDS Patient Care STDs. 2015;29(8):423–29. 26083143 10.1089/apc.2014.0303 PMC4601552

[30] Smith DK, Van Handel M, Wolitski RJ, Stryker JE, Hall HI, Prejean J, et al Vital signs: estimated percentages and numbers of adults with indications for preexposure prophylaxis to prevent HIV acquisition - USA, 2015. MMWR Morbidity Mortality Weekly Rep. 2015;64(46):1291–95. 10.15585/mmwr.mm6446a4 26606148

[31] GMHC Is PrEP or PEP for you?: gay mens health crisis. 2016 Available from: http://www.gmhc.org/prep

[32] Fatch R, Emenyonu NI, Muyindike W, Kekibiina A, Woolf-King S, Hahn JA Alcohol interactive toxicity beliefs and ART non-adherence among HIV-infected current drinkers in Mbarara, Uganda. AIDS Behav. 2016 [Epub ahead of print]. 10.1007/s10461-016-1429-3 PMC5116271 27198557

[33] Pellowski JA, Kalichman SC, Kalichman MO, Cherry C Alcohol-antiretroviral therapy interactive toxicity beliefs and daily medication adherence and alcohol use among people living with HIV. AIDS Care. 2016;28(8):963–70. 26964014 10.1080/09540121.2016.1154134 PMC4963817

[34] Jalbert JJ, Quilliam BJ, Lapane KL A profile of concurrent alcohol and alcohol-interactive prescription drug use in the US population. J Gen Intern Med. 2008;23(9):1318–23. 18575940 10.1007/s11606-008-0639-4 PMC2518026

[35] Horne R, Buick D, Fisher M, Leake H, Cooper V, Weinman J Doubts about necessity and concerns about adverse effects: identifying the types of beliefs that are associated with non-adherence to HAART. Int J STD AIDS. 2004;15(1):38–44. 14769170 10.1258/095646204322637245

[36] Sweeney SM, Vanable PA The association of HIV-related stigma to HIV medication adherence: a systematic review and synthesis of the literature. AIDS Behav. 2016;20:29–50. 26303196 10.1007/s10461-015-1164-1

